# The Synergistic Impact of Polyphenols on Collagen Fiber–Starch Composite Films for Enhanced Physical Integrity and Antioxidant Capacity

**DOI:** 10.3390/foods15030549

**Published:** 2026-02-04

**Authors:** Jiapeng Li, Jing Xu, Wenjian Cheng, Hong Jin

**Affiliations:** 1 School of Biological Engineering, Dalian Polytechnic University, Dalian 116034, China; 19819205955@163.com; 2 College of Food Science, Fujian Agriculture and Forestry University, Fuzhou 350002, China; xuj_june@163.com

**Keywords:** edible films, polyphenols, collagen fiber–starch composite, antioxidant properties, food packaging

## Abstract

Edible films are increasingly recognized as promising sustainable packaging alternatives, but often face challenges such as poor mechanical strength, limited barrier properties, and low oxidative stability. This study aimed to enhance the physicochemical performance of collagen fiber–starch composite films by incorporating polyphenols (including tannic acid (TA), caffeic acid (CA), and their oxidized forms, OTA and OCA) as natural cross-linkers and antioxidants. Results showed that the addition of 0.1% TA increased the tensile strength by approximately 45% compared to the control, while simultaneously reducing the water vapor permeability from 1.32 to 1.26 g·mm/kPa·h·m^2^, with TA outperforming CA due to its higher molecular weight and stronger intermolecular interactions. Oxidized polyphenols further improved the mechanical and water vapor barrier properties via quinone-induced covalent cross-linking, thereby forming a denser film network. The films also exhibited enhanced UV–visible light shielding, with nearly complete ultraviolet blockage (transmittance is close to zero in the 200–280 nm range). Non-oxidized polyphenols showed higher antioxidant activity in the ABTS and reducing power assays, while release kinetics analysis revealed the highest release rate in 50% ethanol, indicating a pronounced solvent-dependent behavior. Specifically, films with 0.1% TA exhibited an ABTS radical scavenging activity of over 80%, significantly higher than the control. Overall, polyphenols effectively improve film performance through cross-linking and structural modification, offering a theoretical foundation for designing active packaging for targeted food systems.

## 1. Introduction

Edible films have attracted considerable interest as a promising and environmentally friendly alternative to conventional petrochemical-based packaging materials [1,2]. These films, derived from natural compounds, for example, proteins and polysaccharides, offer a range of benefits including biodegradability, biocompatibility, and the capacity to improve the nutritional profile of food products [3,4,5,6]. Despite their advantages, current edible films, particularly those based on starch and proteins, face limitations such as poor flexibility and barrier properties in starch-based films and high production costs for protein-based films. To address the challenges, the incorporation of natural polymers, nanocomposites, and edible coatings has been explored to improve their functional properties.

In the context of collagen stabilization, various physical and chemical approaches beyond polyphenols have been reported, such as glutaraldehyde cross-linking [7], enzymatic cross-linking [8], and treatment with natural aldehydes (e.g., vanillin) [9]. These methods enhance the stability of the collagen network by introducing covalent cross-links; however, the use of some cross-linking agents raises concerns regarding their biocompatibility or safety. In contrast, polyphenols exhibit distinct advantages for collagen stabilization due to their natural origin and favorable safety profile. For example, tannic acid can bind to collagen fibers via multiple hydrophobic interactions and hydrogen-bonding, thereby enhancing thermal stability and mechanical properties of collagen-based materials [10]. Polyphenols have been extensively studied for their potential as natural cross-linking agents and antioxidants in protein-based films [4,11,12]. The mechanism of action of polyphenols entails hydrogen bonds, hydrophobic interactions, and covalent bond linkages with protein matrix amino acid residues, leading to an improved film structure with increased rigidity and reduced permeability [13,14,15]. Moreover, their antioxidant properties contribute to extending the shelf life of packaged food by inhibiting lipid oxidation and other oxidative processes. The antioxidant activity of polyphenols stems from their capacity for electron donation and free radical scavenging, thereby inhibiting the oxidation of lipids and other biomolecules. The antioxidant action of polyphenols stems principally from their phenolic hydroxyl groups. These groups can donate hydrogen atoms or electrons to quench reactive oxygen species (ROS), thus disrupting oxidative chain reactions [16]. This property highlights their significant potential for use in active packaging systems. For instance, studies have shown that incorporating polyphenols such as tea polyphenols, catechins, or gallic acid into gelatin [17], chitosan [18], or starch-based [19,20] films not only enhances the physicochemical properties of the films but also markedly improves their free radical scavenging capacity and reducing power, thus effectively delaying the oxidative deterioration of packaged foods [21].

The present work seeks to mitigate the shortcomings of current edible films by utilizing polyphenols as natural cross-linking and antioxidant agents. Through a systematic investigation of the efficacy of polyphenol type and concentration in enhancing the physical barrier and antioxidant activity of composite films, this research elucidates key structure–property relationships. These findings provide valuable insights into formulation and processing conditions for optimizing the performance of polyphenol-incorporated composite films as food packaging materials. The findings suggest that polyphenols bolster the mechanical strength, antioxidant activity, and barrier performance of composite films, rendering them applicable to a diverse spectrum of food products [17,19]. Additionally, the work offers a better understanding of how the release of polyphenols from composite films can be regulated based on the food environment, which enables the design of food packaging that releases antioxidants preferentially in conditions that mimic the presence of ethanol, such as in chilled or fermented foods. The research makes a significant contribution to the progress in sustainable and packaging materials by demonstrating the prospects of polyphenols as natural agents for improving the properties of composite films. The findings have the potential to inspire further investigations and applications in edible film technology and promote the advancement of the food packaging industry towards more sustainable practices.

## 2. Materials and Methods

### 2.1. Materials

Dry *Gadus chalcogrammus* skin was procured from Jilin, China. Corn starch (food grade) was supplied by Linghua Group Co., Ltd. (Jining, China). Glycerol (Analytical Reagent, AR), acetic acid (AR), NaOH (AR), and 2,2-Diphenyl-1-pikryl-hydrazyl (DPPH, AR) were obtained from Sinopharm Chemical Reagent (Shanghai, China). Tannic acid (AR), caffeic acid (AR), and ABTS reagent (AR) were purchased from Macleans Biochemical Technology Co., Ltd. (Shanghai, China). DPPH was obtained from Sigma-Aldrich, St. Louis, MO, USA.

### 2.2. Film Preparation

#### 2.2.1. Isolation of Collagen Fibers from Fish Skin

Fish skins were soaked in water for one hour to remove fish scales and other impurities. The cleaned skins were then treated with 0.1 M NaOH (1:10 *w*/*v*) at 4 °C for 4 h to eliminate soluble proteins and pigments. Subsequently, the skins were rinsed with tap water until the pH approached 7.0. The skins were further treated with 0.05 M HCl (1:10 *w*/*v*) at 4 °C for 6 h, homogenized using a high-speed pulverizer (XHF-DY, Unite Mechanical Manufacturing Co., Ltd, Suzhou, China), and filtered through a 45-mesh sieve. The filtrate was washed three times with deionized water and centrifuged. The resulting precipitate was used as the insoluble collagen fibers.

#### 2.2.2. Fabrication of Collagen Fiber–Starch Composite Films

A range of diverse concentrations (0, 10, 15, 20, and 25 mg/mL) of tannic acid (TA) and caffeic acid (CA) solutions were prepared by dissolving these polyphenols in deionized water. Non-oxidized polyphenol solutions were shielded from light, while oxidized polyphenol solutions (oxidized tannic acid and oxidized caffeic acid) were prepared by adjusting the pH to 9 with 1 M NaOH and exposing the solutions to air for 12 h under continuous stirring. The film-forming dispersion was prepared as follows: starch was dispersed in deionized water, and glycerol was added at 30% (*w*/*w*) relative to the starch mass (i.e., 1.2 g glycerol per 4 g starch). The mixture was heated in a 90 °C water bath under continuous stirring for 30 min to obtain a homogeneous 4% (*w*/*w*) starch paste. Subsequently, the starch paste was cooled to approximately 50 °C, and predetermined amounts of the starch paste and collagen fiber suspension were combined and homogenized to obtain 100 g of a collagen fiber–starch mixture. In the final blend, the starch concentration was fixed at 4% (*w*/*w*) of the total mass, and collagen fibers (on a dry-weight basis) accounted for 10.0% (*w*/*w*) of the starch mass. Finally, the dispersions were cast onto petri dishes and air-dried at room temperature for 24 h.

### 2.3. Mechanical Property Assessment

The tensile strength (TS) and elongation at break (EAB) of the films were evaluated using a texture analyzer (EZ TEST, Shimadzu, Kyoto, Japan) according to the method described by Susmitha [22], with slight modifications. Film samples were cut into 10 × 50 mm strips and stored in a constant temperature and humidity chamber (SPX-250, Shanghai Xuanyi Instrument Co., Ltd., Xuanyi, Shanghai, China) at 25 °C and 50 ± 3% RH for 72 h before testing. The initial gauge length was set to 50 mm, and the strips were stretched at a constant crosshead speed of 50 mm/min until fracture. Each sample was tested in at least five replicates, and the results were reported as mean values. TS and EAB were calculated using the equation below.

$$TS\;(MPa)=\frac{F}{A}$$

$$EAB\;(\%)=\frac{L_{2}-L_{1}}{L_{1}}\times 100$$

F is the maximum load (N) needed to stretch the composite film to its breaking point. A is the cross-sectional area (m^2^) of the film. TS is the tensile strength of the film, typically expressed in units of pressure, such as MPa. L_1_ is the initial grip separation (30 mm). L_2_ is the length at break.

### 2.4. Water Vapor Permeability Measurement

The water vapor permeability (WVP) was determined according to the method described [23]. Film samples were equilibrated at 25 °C and 50% RH for 72 h before testing. The WVP test was conducted under the same constant conditions of 25 °C and 50% RH using the desiccant method. The WVP was calculated using the following equation:

$$WVP\;(g\cdot mm/kPa\cdot h\cdot m^{2})=\frac{\Delta m\times L}{t\times S\times \Delta P}$$

Δm is the mass of water vapor (g) transmitted through the film and absorbed by the desiccant over the time interval t. L is the film thickness (mm). t is the time interval (h). S is the exposed area of the film (m^2^). ΔP is the partial water vapor pressure difference (kPa) across the two sides of the film. Each film was performed in triplicate.

### 2.5. Water Solubility Determination

The water solubility (Ws) was estimated according to the method described by Qiao [24], with some modifications. The water solubility of the film was calculated using the following equation:

$$Ws\;(\%)=\frac{W_{1}-W_{2}}{W_{1}}\times 100$$

W_1_ is the initial dry weight of the film. W_2_ is the weight of the undissolved fraction after immersing the film in deionized water. Ws is the water solubility of the film, expressed as a percentage.

### 2.6. Light Transmission and Opacity Analysis

The light transmission and opacity of the films were determined according to the method described previously [25]. The absorbance of the film at 600 nm was measured using a UV–visible spectrophotometer (UV-1800, Shimadzu, Kyoto, Japan). The opacity was evaluated based on the absorbance of the film at 600 nm using the equation below.

$$Opacity=\frac{A_{600}}{x}$$

where A_600_ is the absorbance of the film at 600 nm, and x is the film thickness (mm). Results are expressed as A mm^−1^. This formula transforms absorbance into opacity, where higher values of opacity indicate lower transparency.

### 2.7. Microstructural Examination by Scanning Electron Microscopy (SEM)

Film samples were dried in a desiccator containing silica gel at 25 °C for 2 weeks before testing. Film samples were broken in liquid nitrogen to obtain a clean break. The broken samples were then fixed on the sample stage of the SEM. Gold sputtering was performed on the sample surfaces for 2 min. SEM micrographs were obtained using a scanning electron microscope (Nova Nano SEM 450, FEI Company, Hillsboro, OR, USA), operating at an accelerating voltage of 5.0 kV. This voltage setting is chosen based on the properties of the sample and the desired imaging resolution. SEM images were taken of both the surface and cross-section of the pre-dried film samples.

### 2.8. Thermal Analysis by Differential Scanning Calorimetry (DSC)

The thermal properties of the films were analyzed using a differential scanning calorimeter (DSC200F3, NETZSCH, Selb, Germany). Film samples were desiccated in a silica gel-filled desiccator at 25 °C for two weeks to remove moisture before testing. Approximately 4 mg of the pretreated samples was sealed in aluminum pans, and the temperature scan was conducted from 20 °C to 300 °C at a heating rate of 10 °C/min. An empty aluminum pan served as the reference. The heat flow curves were analyzed using the instrument’s proprietary software, and data from the second heating scan were reported to minimize the effects of thermal history.

### 2.9. Characterization by Fourier Transform Infrared Spectroscopy (FTIR)

FTIR spectra of the film samples were obtained using a VERTEX 70 FTIR Spectrometer (Bruker Co., Ettlinger, Germany) equipped with a horizontal ATR Trough plate crystal cell, following the method described [26]. Samples were desiccated in a silica gel-filled desiccator for two weeks before testing. Spectra were recorded from 650 to 4000 cm^−1^ in 32 scans with a resolution of 4 cm^−1^. Background spectra were obtained from a clean cell without samples.

### 2.10. Evaluation of Antioxidant Capacity

Each 50 mg of pretreated film sample was ground and immersed in 50 mL of distilled water, and then centrifuged at 5000 rpm for 10 min at room temperature. The supernatant containing the extract was used for antioxidant activity analysis. The antioxidant activities of the films were determined by the reducing capacity and the ABTS radical cation decolorization assay.

#### 2.10.1. Assessing Reducing Power

The reducing capacity of the films was assessed using the method described with minor modifications [27]. A mixture of 100 μL of sample solution, 100 μL of 0.2 M PBS (pH 6.6), and 100 μL of potassium ferricyanide solution (1% *w*/*v*) was incubated in a water bath at 50 °C for 20 min and then rapidly cooled to room temperature. Immediately, trichloroacetic acid (TCA) solution (100 μL, 10% *w*/*v*) was added to the film extract. If precipitation occurred, the solution was centrifuged at 6400 rpm for 10 min. Then, 150 μL of supernatant was mixed with 150 μL of distilled water and 30 μL of FeCl_3_ solution. The mixture was allowed to stand at room temperature for 10 min before recording the absorbance at 700 nm using a Microplate reader (Spectra MAX Plus, Molecular Devices, Sunnyvale, CA, USA). The absorbance value of the sample suggested its reducing power. Each film was tested in triplicate.

#### 2.10.2. Quantifying ABTS Radical Scavenging Activity

The ABTS free radical scavenging rate of the films was measured using the method described [28]. Initially, the ABTS·^+^ radical solution was generated by combining 7 mM aqueous ABTS stock solution with 2.45 mM potassium persulfate solution and incubating under ambient conditions in the dark for 12–16 h. Then, this mixed solution was adjusted 48 to 50 times using distilled water to achieve a 0.70 ± 0.02 absorbance value at 734 nm. Then, the ABTS free radical scavenging rate of the films was measured by adding 30 μL of the sample extract solution to 270 μL of the diluted ABTS·^+^ solution. After 6 min, the absorbance of the mixture was recorded at 734 nm using a Microplate reader (Spectra MAX Plus, Molecular Devices). Distilled water was used as the blank control. Each film was tested in triplicate. The ABTS free radical scavenging rate was calculated using the equation below.

$$\mathrm{ABTS}\;\mathrm{free}\;\mathrm{radical}\;\mathrm{scavenging}\;\mathrm{rate}\;(\%)=\left(\frac{A_{0}-A_{x}}{A_{0}}\right)\times 100$$

A_0_ is the absorbance of the blank control (distilled water instead of the sample solution). A_x_ is the absorbance of the sample solution.

### 2.11. Polyphenol Release Kinetics from Composite Films

The release of polyphenols from the composite films was investigated based on the methods reported [29]. Ethanol solutions with concentrations of 0%, 20%, 50%, 75%, and 95% were used to simulate different food systems for the release studies. The release results were reported in terms of DPPH radical scavenging activity. Briefly, 50 mg of the film sample was placed in 50 mL of each ethanol solution. The release studies were conducted for 300 min at ambient temperature. At regular intervals, 100 μL of sample solution and 200 μL of 0.1 mM DPPH (prepared with 95% ethanol) were mixed. The absorbance was measured at 517 nm after 30 min in the dark.

$$\mathrm{DPPH}\;\mathrm{radical}\;\mathrm{scavenging}\;\mathrm{activity}(\%)=\left(\frac{A_{0}-A_{X}}{A_{0}}\right)\times 100$$

A_0_ is the absorbance of the blank control (distilled water instead of the sample solution). A_X_ is the absorbance of the sample solution.

### 2.12. Statistical Analysis

SPSS 20.0 software (IBM, New York, NY, USA) was used to analyze data to determine statistical significance. Results are presented as the mean ± standard deviation (SD) of triplicate experiments. To assess the significance of differences among treatment groups, one-way analysis of variance (ANOVA) was performed, followed by Duncan’s multiple-range test. A *p*-value of less than 0.05 was considered statistically significant.

## 3. Results and Discussion

### 3.1. Mechanical and Structural Characteristics of Composite Films

**Figure 1:**
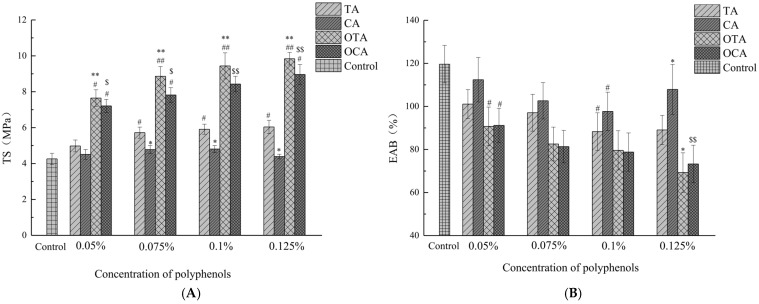
The impact of polyphenol content on the TS (**A**) and EAB (**B**) of composite films. OTA, TA, CA, and OCA denote composite films containing oxidized tannic acid, tannic acid, caffeic acid, and oxidized caffeic acid, respectively. The symbols “#” and “##” represent significance levels compared to the control group, with *p* < 0.05 and *p* < 0.01, respectively. The symbols “*” and “**” represent significance levels compared to TA at the same addition level, with *p* < 0.05 and *p* < 0.01, respectively. The symbols “$” and “$$” represent significance levels compared to CA at the same addition level, with *p* < 0.05 and *p* < 0.01, respectively.

The study on the mechanical properties of starch–collagen fiber composite films with varying concentrations of polyphenols revealed that the control film exhibited the lowest TS and the highest EAB (Figure 1). With the increasing concentrations of TA, CA, OTA, and OCA, both TS and EAB were observed to change correspondingly. This suggests that phenolic compounds interact with the polymer matrix in the film, leading to alterations in the mechanical properties.

The formation of cross-links between phenolic compounds and proteins can result in various bonding interactions, comprising covalent bonds, hydrophobic interactions, and hydrogen bonds [30,31]. Phenolic compounds have the ability to form cross-links between proteins by reacting with multiple sites [31,32,33]. The enhanced interactions between protein molecules contribute to the increased rigidity of the composite films, which in turn reduces their extensibility. Concurrently, the phenolic hydroxyl groups in polyphenol molecules form strong hydrogen-bonding interactions with the hydroxyl groups on starch chains. This interaction restricts the migration and rearrangement of starch chains, thereby promoting the densification of the film network and contributing to enhanced tensile strength and reduced elongation at break [34]. Similarly, Nilsuwan reported increased TS and decreased EAB in gelatin films incorporated with EGCG, attributed to hydrogen bond formation between proteins and phenolic compounds [35]. Interestingly, when the concentration of caffeic acid exceeded 0.1%, a slight decrease in TS and an increase in EAB were observed (Figure 1). This could be due to the excessive amount of caffeic acid acting as a plasticizer in the composite films [36]. This plasticizing effect influences the collagen network as well as the starch continuous phase, partially weakening the hydrogen-bond interactions among starch chains and between starch and polyphenols, thereby reducing film rigidity and partially restoring extensibility. The cross-linking effect of small-molecule phenolic compounds is predominant at low concentrations, whereas at high concentrations, the plasticizing effect becomes dominant. Comparatively, tannic acid played a superior role in enhancing the mechanical behavior of the film compared to caffeic acid, which could be attributed to stronger interactions between tannic acid and collagen fibers. The structural features of polyphenol molecules, such as molecular weight, structural flexibility, and the number of hydroxyl groups, significantly influence the interaction between proteins and phenolic compounds [37,38,39]. The interaction strength between polyphenols and proteins correlates with polyphenol molecular weight [40]. Due to its high molecular weight, tannic acid is capable of forming stronger or more preferred bonds with proteins, thereby significantly enhancing the mechanical properties of composite films [41,42].

Furthermore, the addition of OTA and OCA resulted in greater mechanical properties compared to tannic acid and caffeic acid. Under alkaline pH and oxygen exposure conditions, phenolic compounds can be oxidized to quinones, which act as protein cross-linkers. These quinones can react with amino or sulfhydryl side chains of polypeptides to form covalent C-N or C-S bonds [43,44,45]. Cao reported that the maximum TS of gelatin films incorporated with tannic acid was achieved when the pH value of the film-forming solution was 9.0 [46]. Additionally, OCA contributed to enhancing the TS of the film compared with CA, possibly owing to the formation of C-N covalent bonds with collagen fibers and enhanced interactions between oxidized caffeic acid and collagen fibers. Moreover, the oxidation reactions produce high-molecular-weight phenols and a sufficient number of remaining unreacted phenolic sites, which enhance the degree of protein cross-linking [47,48].

### 3.2. Water Vapor Permeability of the Composite Films

Water vapor permeability is a crucial function of food packaging films, as it helps to block water vapor in the air and slow down the moisture changes of food in packaging. Lower WVP in packaging can better maintain food quality [49]. Our findings indicate that the WVP of composite films without polyphenols (control group) was 1.321 g/kPa·h·m^2^. When the concentration of polyphenol was below 0.1%, the WVP of films with TA or CA gradually decreased, but it showed a slight increase compared to the control when the concentration was over 0.1%.

Notably, the variation in WVP exhibits a significant inverse correlation with the changes in tensile strength (TS) described in Section 3.1. This coupled evolution of mechanical and barrier properties primarily reflects polyphenol-induced alterations in the cross-linking density and compactness of the film matrix. As outlined in Section 3.1, an appropriate polyphenol content (TA or CA concentration < 0.1%) can effectively cross-link with collagen fibers and starch molecules via hydrogen bonding and hydrophobic interactions, forming a compact three-dimensional network. This network not only enhances the TS of the film by strengthening intermolecular interactions but also increases the tortuosity of water diffusion pathways, thereby reducing WVP [50,51]. In the composite system of this study, where starch serves as the continuous phase and collagen fibers function as a reinforcing network, the interaction between polyphenols and the starch matrix is particularly critical. Strong hydrogen bonds are established linking the phenolic hydroxyl groups of polyphenols to the hydroxyl groups on starch chains. This interaction partially conceals the intrinsic hydrophilic hydroxyl groups of starch, thereby limiting the available sites for water adsorption and diffusion. More importantly, this interaction effectively immobilizes starch molecular chains through a molecular pinning effect, reinforcing the continuous-phase matrix and playing a dominant role in improving the overall moisture barrier performance of the film [52]. However, when the polyphenol content exceeds the optimal threshold (e.g., CA > 0.1%), excess polyphenol molecules that do not participate in effective cross-linking may exhibit plasticizing behavior. These molecules disrupt the original hydrogen bonds among starch chains and between starch and collagen, weakening the rigidity of the polymer network and leading to a decrease in TS [53]. Simultaneously, unbound hydrophilic polyphenols may generate localized hydrophilic micro-domains within the film, providing additional adsorption and diffusion sites for water molecules and ultimately resulting in a rebound increase in WVP. This phenomenon is consistent with previous reports describing performance deterioration caused by insufficient or excessive cross-linking. In conclusion, a certain number of phenolic compounds added into composite films can reduce the WVP by enhancing the degree of cross-linking of the film-forming substrate and improving the structure of the film. However, excessive polyphenols in films increase the film’s water-absorbing capacity and accelerate the passage of water molecules through the film.

Notably, oxidized polyphenols (OTA and OCA) exhibited consistently superior WVP barrier performance compared with TA and CA across all tested concentrations (Table 1). This advantage is primarily attributed to the quinone moieties of oxidized polyphenols, which enable the formation of stronger covalent C-N bonds with amino groups in collagen, resulting in a more extensive and robust cross-linking network [54]. This enhanced cross-linking further consolidates the starch–collagen fiber interface. Moreover, additional interactions and potential secondary interactions between quinones and starch likely promote a more homogeneous and compact continuous phase, thereby significantly inhibiting water vapor permeation. In addition, oxidized polyphenols optimize the hydrophilic–lipophilic balance of the starch matrix, facilitating the formation of a denser film structure with reduced porosity and improved uniformity, which effectively impedes water vapor diffusion [55]. These mechanisms are fully consistent with the optimal WVP barrier performance observed in films containing oxidized polyphenols.

### 3.3. Water Solubility of the Composite Films

Solubility and swelling are commonly used to evaluate the intermolecular interactions of film-forming components [56]. The solubility of the composite film without phenolic substances (control group) was 16.32%. The addition of polyphenol, regardless of its content, caused a decrease in film solubility compared with the control. The solubilities of composite films decreased significantly with increasing concentrations of TA, OTA, or OCA. This suggests that the molecular activities within the film-forming components were constrained due to the cross-linking effect of polyphenols when added to the films, and the solubilities were reduced.

However, when the caffeic acid levels were higher than 0.05%, an increase in solubility was observed, but it was still lower than that of the control group. These results indicate that caffeic acid at concentrations over 0.05% could be an excessive addition that introduces abundant hydrophilic groups, increasing the hydrophilicity and solubility of the composite films.

### 3.4. Light Transmission and Opacity

A film’s optical performance plays a pivotal role in market success by directly affecting product visual appeal, thus constituting a key functional requirement [57]. The transparency of the composite films was found to be affected by the types and amounts of polyphenols incorporated (Table 1). When the TA and CA levels were less than 0.05% and 0.075%, respectively, the opacity was not statistically different from the control film (*p* > 0.05). In contrast, when the concentrations exceeded these thresholds, a marked rise in opacity was detected (*p* < 0.05). This can be ascribed to the formation of strong cross-links between collagen fibers in the presence of tannic acid and caffeic acid, leading to increased turbidity and opacity.

**Figure 2:**
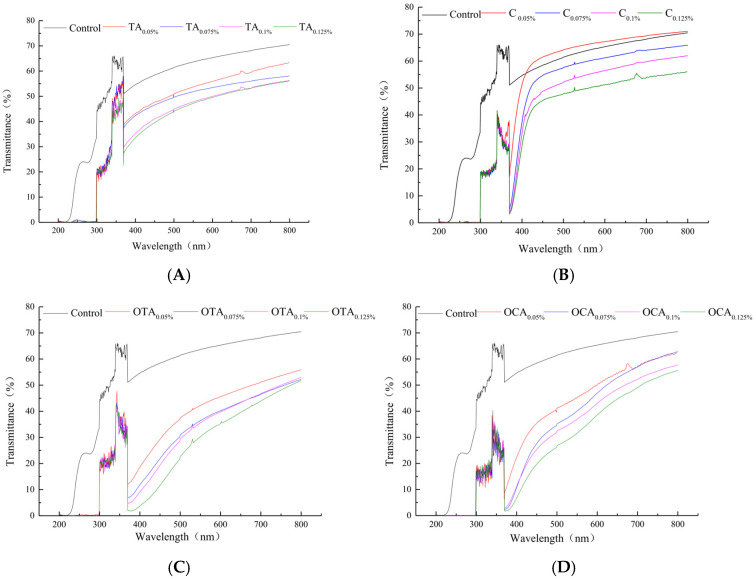
Influence of polyphenol type on the transmittance of composite films: (**A**) Tannic acid (TA), (**B**) caffeic acid (CA), (**C**) oxidized tannic acid (OTA), and (**D**) oxidized caffeic acid (OCA). The shaded areas indicate the UV-C (200–280 nm) and UV-B (280–400 nm) regions. The instrument’s lower sensitivity below 250 nm contributes to the observed noise but does not affect the central conclusion of negligible UV transmission.

The films’ opacity rose markedly with the oxidized polyphenols levels, surpassing that of TA- or CA-containing films. This is primarily attributed to the formation of colored quinones following the oxidation of polyphenols, which substantially enhanced the films’ opacity. Preventing food from the effects of light, particularly ultraviolet radiation, is a desired characteristic of packaging materials [58]. Light, especially in the UV range (below 400 nm), is a major factor promoting fat oxidation in food [59]. Figure 2 shows the UV-Vis transmittance of the composite films. Despite some instrumental noise noted in the deep UV region (particularly below 250 nm), which is a known sensitivity limitation of the silicon-based detector in the spectrophotometer used (UV-1800, Shimadzu Corporation, Kyoto, Japan), the key trend is unequivocal. A decrease in transmission was observed across all films enriched with polyphenols, compared to the control film. Critically, within the entire UV-C region (200–280 nm) responsible for the most severe photo-oxidation, all modified films exhibited near-zero transmittance, constituting an excellent barrier. In particular, in the 200–280 nm range, the transmittance of the composite film was almost zero, indicating an excellent barrier against light-induced damage, especially from UV. This observation is consistent with findings reported by Cassar, J.R. et al. (2020), which could result from the strong absorption capacity of benzene rings and carbonyl groups in polyphenols for n → π* transitions in the UV range [59].

Transmittance in the visible light range (400–800 nm) decreased as more polyphenol was added to the composite films. Films with OTA and OCA showed better light barrier properties than those with TA and CA. Notably, the observed changes in transmittance and opacity reflect not only optical performance but also underlying microstructural evolution. The markedly reduced transmittance and increased opacity of films containing oxidized polyphenols (OTA and OCA) in the UV–Vis region, particularly between 400 and 600 nm, are attributed to the strong light absorption of their quinone structures. This optical behavior originates from enhanced interactions between oxidized polyphenols and collagen fibers. As discussed in Section 3.1 and Section 3.2, quinones formed during oxidation can generate covalent C–N linkages with amino groups in collagen, resulting in a denser and more interconnected cross-linked network. This densified structure not only improves mechanical strength and moisture resistance but also increases light-scattering interfaces and absorption sites, thereby manifesting macroscopically as reduced transmittance and increased opacity [20]. Consequently, the distinct optical characteristics of films containing oxidized polyphenols can be regarded as macroscopic evidence of stronger molecular interactions and network formation.

### 3.5. Scanning Electron Microscopy Analysis of the Composite Films

**Figure 3:**
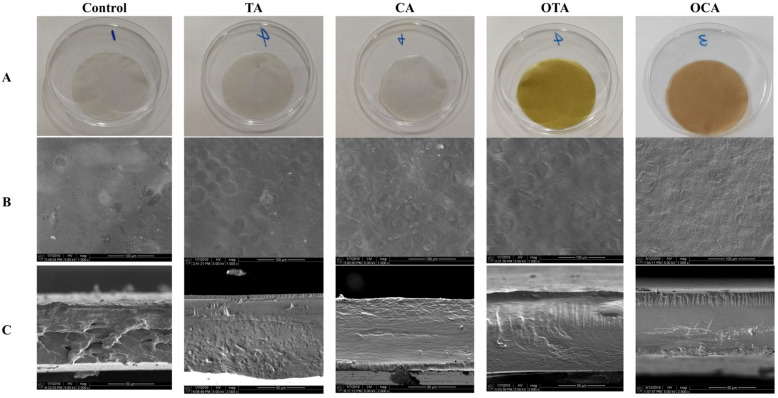
Microstructural images of composite films. TA, CA, OTA, and OCA represent composite films containing tannic acid, caffeic acid, oxidized tannic acid, and oxidized caffeic acid, respectively. (**A**) Macroscopic surface, (**B**) microscopic surface (1000×), and (**C**) microscopic cross-section (2000×).

Figure 3 depicts the scanning electron images of the film surface and cross-section incorporating various polyphenols at 0.1% and of the control group. Pores were observed in all films, likely caused by the free evaporation of water during drying. The surface and cross-section of the films appeared smoother with the addition of polyphenols, especially with OTA and OCA, compared to the control. These findings suggest polyphenol addition resulted in improved compatibility and a more compact film structure.

The improved film morphology is attributed not only to enhanced intermolecular interactions between polyphenols and collagen fibers [43] but also to their effective integration with the starch matrix. Polyphenol molecules, particularly through their phenolic hydroxyl groups, form a dense hydrogen-bonding network with hydroxyl groups on starch chains, which reduces interfacial defects, suppresses phase separation, and promotes a more uniform and compact microstructure [19,20]. Oxidized polyphenols (OTA and OCA), owing to higher reactivity and potential covalent cross-linking, exert a stronger restraining and integrating effect on starch chains, which explains why films containing them display the highest surface smoothness and structural compactness.

### 3.6. Thermal Stability and Glass Transition Temperature of the Composite Films

**Figure 4:**
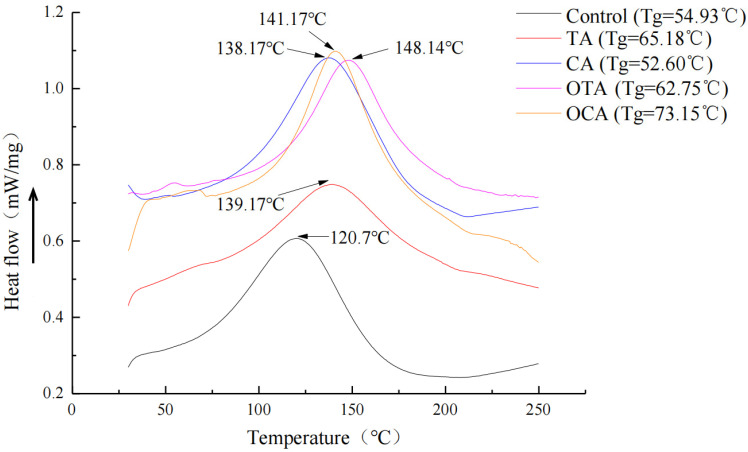
DSC curves of composite films containing different polyphenols. TA, CA, OTA, and OCA denote composite films containing tannic acid, caffeic acid, oxidized tannic acid, and oxidized caffeic acid, respectively. The arrow indicates the position of the glass transition temperature (Tg) corresponding to each sample on the DSC curve.

The Tg, which reflects the movement of molecules within the film components, is closely associated with the molecular structure of the composite film components. When polymer molecular movement is restricted, the Tg increases. As depicted in Figure 4, two major thermal transitions were identified over the temperature range of 30–250 °C. The Tg was determined from the step change in the DSC baseline using the tangent method, yielding values of 54.93 °C (control), 65.18 °C (TA), 52.60 °C (CA), 62.75 °C (OTA), and 73.15 °C (OCA). Notably, the OCA and OTA films exhibited markedly higher Tg values than the control, indicating effective restriction of polymer chain mobility, particularly induced by oxidized caffeic acid. This behavior is attributed to the formation of a denser three-dimensional network between collagen fibers and the starch matrix, arising from hydrogen bonding and possible covalent cross-linking introduced by polyphenols, especially in their oxidized forms. A second distinct endothermic event was observed in the range of 120–148 °C, which is assigned to the evaporation of residual bound water and/or collagen denaturation, rather than a glass transition. For films containing oxidized polyphenols (OTA, OCA), the peak temperature (Tp) of this endotherm shifted toward higher values, suggesting that stronger polyphenol–collagen interactions (e.g., quinone–amine cross-linking) enhance collagen structural stability and/or modify the binding state of water molecules [60]. The DSC analysis was designed to probe thermal transition behavior relevant to material processing and application, thereby elucidating underlying structural changes. Although thermal decomposition was not assessed, the observed Tg elevation—particularly in the CA and OTA films—provides direct evidence of network densification and restricted molecular motion induced by polyphenol cross-linking. These findings are mechanistically consistent with the enhanced tensile strength and reduced water vapor permeability discussed in Section 3.1 and Section 3.2. Therefore, the DSC results offer critical thermodynamic evidence supporting the role of oxidized polyphenols in improving the overall physical performance of the composite films [44].

### 3.7. Analysis of Molecular Interactions by FTIR Spectroscopy

**Figure 5:**
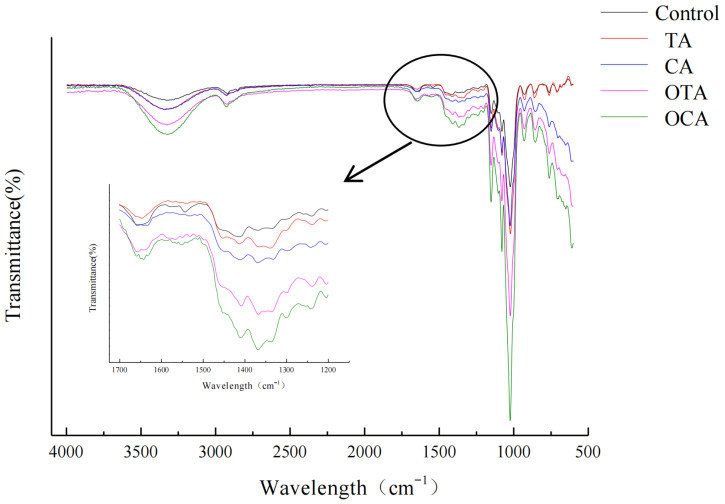
Infrared spectra of composite films with different polyphenol types. TA, CA, OTA, and OCA represent composite films with tannic acid, caffeic acid, oxidized tannic acid, and oxidized caffeic acid, respectively.

As shown in Figure 5, the amplitude of the amide I of composite films with OTA and OCA was larger than that with TA and CA. This finding agrees with Nuthong, who noted that a higher amplitude in the amide I occurred in OCA film than in CA film because adding OCA led to enhanced cross-linking of proteins [61]. Alvarez proved that oxidized caffeic acid promoted gelatin cross-linking by forming C-N covalent bonds with side chains of gelatin using nuclear magnetic resonance technology [47]. Therefore, adding OCA and OTA may promote the formation of covalent bonds between collagen fibers.

The FTIR spectra further confirm the interactions between the polyphenols and the film-forming components. The changes in the amide I and amide II peaks suggest alterations in the secondary structure of the proteins within the films, which could be attributed to the cross-linking reactions. The shift to higher wavenumbers observed in the amide A region suggests that the films have a higher degree of hydrogen bonding, which is a result of the polyphenols interacting with the amino groups of the proteins. Concurrently, characteristic infrared absorption bands of the starch matrix underwent significant changes, providing direct evidence for polyphenol–starch interactions. The O–H stretching vibration band in the range of 3200–3500 cm^−1^ broadened and shifted to lower wavenumbers upon polyphenol addition (e.g., from ~3330 cm^−1^ to 3305 cm^−1^), indicating the formation of new, stronger hydrogen bonds between phenolic hydroxyl groups of polyphenols and hydroxyl groups on starch chains, which weakens the intrinsic O–H bond strength of starch molecules [20]. Furthermore, absorption bands in the 1000–1150 cm^−1^ region, assigned to C–O–C and C–O–H stretching vibrations of starch, displayed altered intensity and band shapes in the presence of polyphenols, further confirming close physical interactions and potential binding between the starch backbone and polyphenol molecules [19]. These spectral changes collectively demonstrate that polyphenols not only cross-link with collagen fibers but also effectively interact with and modify the starch matrix via hydrogen bonding. In conclusion, the FTIR results provide a molecular-level understanding of the interactions that occur when polyphenols are incorporated into composite films. These interactions alter the film structure, ultimately governing their physical and barrier performance. The enhanced cross-linking observed with oxidized polyphenols suggests that these compounds can be particularly effective in improving the overall performance of composite films for food packaging applications.

### 3.8. Antioxidant Activity and Free Radical Scavenging of the Composite Films

**Figure 6:**
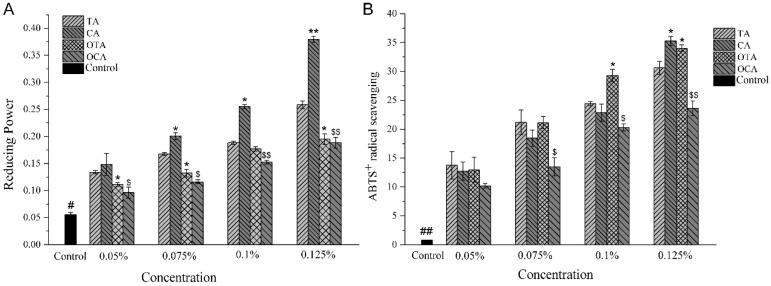
Antioxidant properties of composite films with varying polyphenol types. TA, CA, OTA, and OCA denote composite films containing tannic acid, caffeic acid, oxidized tannic acid, and oxidized caffeic acid, respectively. (**A**) Reducing power; (**B**) ABTS^+^ radical scavenging. In (**A**), “#” denotes a significant difference (*p* < 0.05) from the control group. The symbols “*” and “**” correspond to *p* < 0.05 and *p* < 0.01, respectively, when compared to the TA group at the same addition level. Additionally, symbols “$” (*p* < 0.05) and “$$” (*p* < 0.01) denote significant differences vs. the CA group at the same addition level. In (**B**), “##” denotes a highly significant difference vs. the control at *p* < 0.01. The symbol “*” corresponds to *p* < 0.05 vs. the TA at the same addition level. Furthermore, the symbols “$” and “$$” represent significant differences at *p* < 0.05 and *p* < 0.01, respectively, when compared to the CA group at the same addition level.

The composite films exhibited varying degrees of antioxidant activity, as measured by both the ABTS free radical scavenging assay and the reducing power assay. As shown in Figure 6, the results from these assays were found to be closely correlated, indicating a consistent pattern of antioxidant behavior across different experimental conditions. The ABTS assay measures the capacity of a sample to scavenge free radicals by quantifying the decrease in absorbance of a colored ABTS solution upon reaction with the sample. The reducing power assay, on the other hand, evaluates the ability of a sample to donate electrons by measuring the change in absorbance of a ferricyanide/ferrocyanide solution after the addition of the sample. Both assays are widely used to assess the antioxidant potential of various compounds. In this study, the addition of polyphenols to the composite films led to a significant increase in their antioxidant activity, as evidenced by the higher ABTS and reducing power values compared to the control film without polyphenol addition. This enhancement in antioxidant activity can be attributed to the formation of stable complexes between the polyphenols and collagen fibers in the film matrix, which not only facilitates the scavenging of free radicals but also protects the polyphenols from degradation, thereby maintaining their antioxidant capacity.

The higher ABTS and reducing power values observed for the films with TA and CA compared to those with OTA and OCA suggest that the non-oxidized forms of these compounds are more effective in enhancing the antioxidant properties of the composite films. This could be due to the reduced quenching ability of the oxidized forms, which may form fewer stable complexes with collagen fibers or have a more compact film structure that hinders the release of antioxidant components. Furthermore, the starch matrix, as the continuous phase of the film, plays a critical role in the retention and controlled release of polyphenols [62], primarily through hydrogen bonding and the restriction effect of its three-dimensional network. Starch chains constrain polyphenol molecules via hydrogen bonding and related interactions, influencing their dispersion and accessibility within the matrix. During release, the swelling of starch in ethanol–water solutions alters network pore size, thereby modulating the diffusion path and release rate of polyphenols [63]. The affinity between polyphenols and starch, including hydrogen-bond strength, further governs their dissociation from the matrix. Consequently, the release kinetics of polyphenols in ethanol solutions are determined by the combined effects of starch network swelling and polyphenol–starch interactions. Furthermore, the release study of composite films in various ethanol solutions provided insights into the solvent-dependent release behavior of polyphenols. The higher release rates observed in lower ethanol concentration solutions (20% and 50%) compared to distilled water suggest that ethanol can act as a solvent that facilitates the release of antioxidants from the composite films. This is particularly important in food systems where ethanol concentrations can vary, affecting the release of antioxidants and potentially enhancing the shelf life of the packaged product. In conclusion, the integrated analysis of ABTS and reducing power assays provides a comprehensive understanding of the antioxidant activity of composite films. The results indicate that the type and concentration of polyphenols play a crucial role in determining the antioxidant properties of the films. The release kinetics study further elucidates the environmental factors influencing the release of antioxidants, which is essential for the development of functional food packaging materials that can extend product shelf life and ensure food safety.

### 3.9. Release Kinetics of Polyphenols in Various Ethanol Concentrations

**Figure 7:**
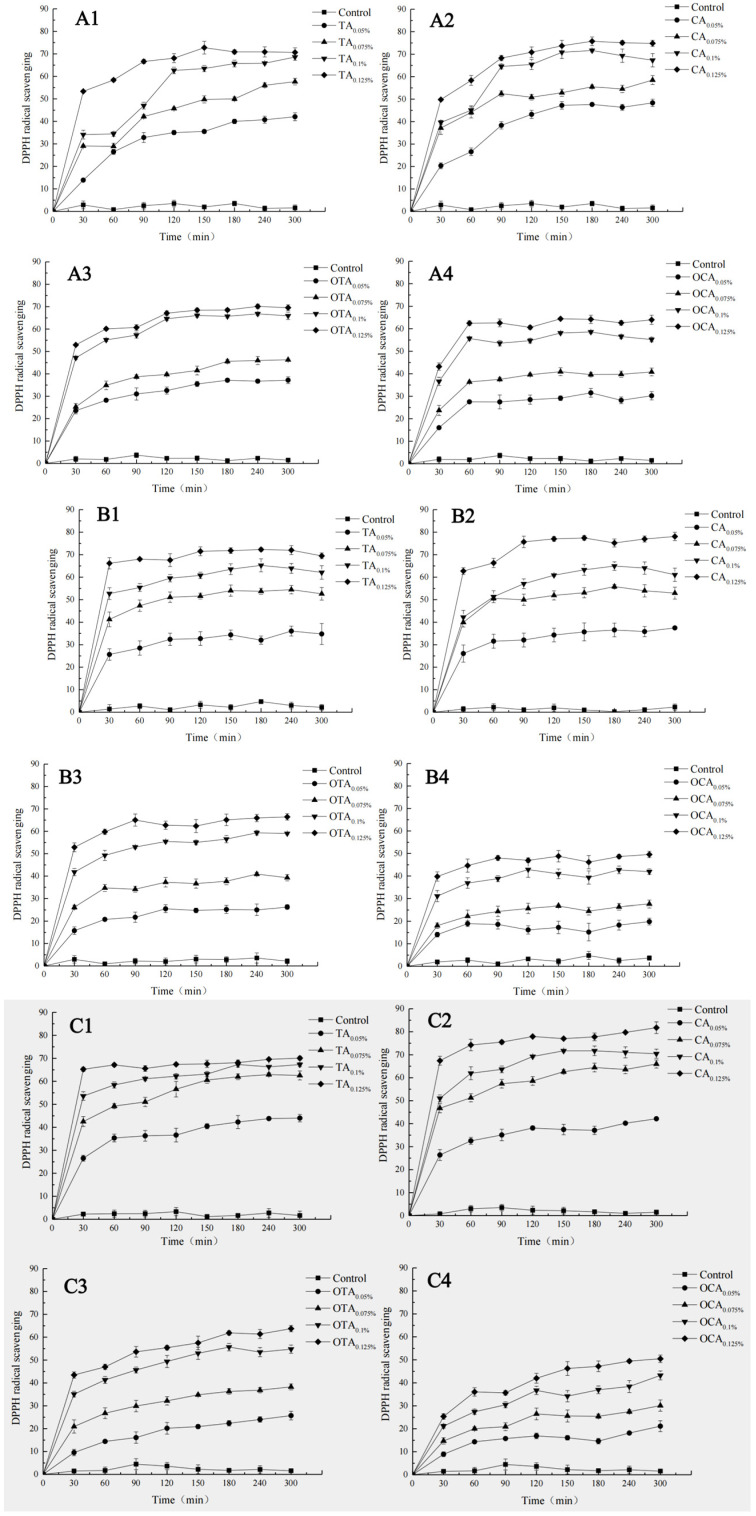

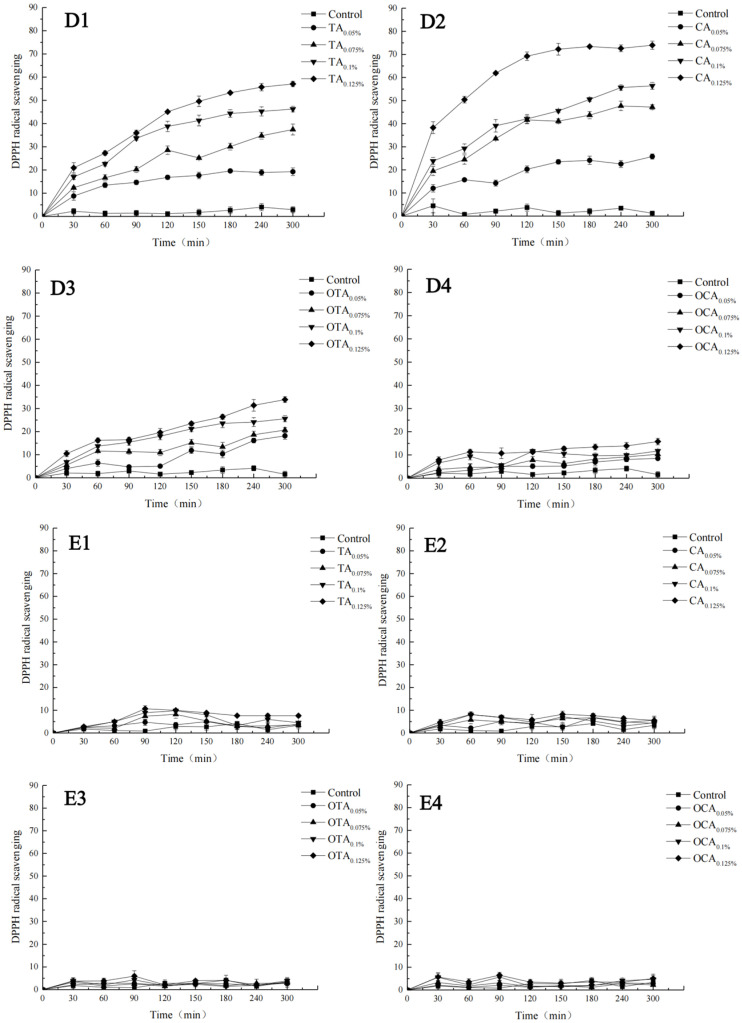
Release kinetics of polyphenols from composite films in different alcohol concentrations: (**A1**–**A4**) 0%; (**B1**–**B4**) 20%; (**C1**–**C4**) 50%; (**D1**–**D4**) 75%; (**E1**–**E4**) 95%.

As shown in Figure 7, the release kinetics of polyphenols from composite films in various ethanol concentrations (0%, 20%, 50%, 75%, and 95%) were systematically investigated to understand the solvent-dependent release behavior and its implications for food packaging applications.

The release of polyphenols from the composite films was found to be significantly influenced by the ethanol concentration. In general, the release rate and amounts of polyphenols increased with increasing ethanol concentration up to 50%, indicating that ethanol can act as a solvent that facilitates the release of antioxidants from the composite films. This is particularly relevant in food systems where ethanol concentrations can vary, affecting the release of antioxidants and potentially enhancing the shelf life of the food product.

However, at 75% and 95% ethanol concentrations, a notable reduction in the release rate and amounts of polyphenols was observed. This could be attributed to the solvent’s high polarity and low water content, which leads to a more hydrophobic environment that restricts the hydration of the composite film and the weakening of the film network. As a result, the polyphenols become less accessible, and their release is impeded. The differential release behavior observed across different ethanol concentrations highlights the potential for controlled release of polyphenols based on the food environment. This could be particularly useful in designing food packaging systems that release antioxidants preferentially in conditions that mimic the presence of ethanol, such as in chilled or fermented foods. In summary, the integrated analysis of the release kinetics in different ethanol concentrations provides valuable insights into the solvent-dependent release behavior of bioactive compounds from composite films. This knowledge can be leveraged to develop functional food packaging solutions that can release antioxidants in a controlled manner, depending on the food environment, thereby extending product shelf life and ensuring food safety.

## 4. Conclusions and Future Perspectives

This study systematically investigated the effects of tannic acid (TA), caffeic acid (CA), and their oxidized derivatives (OTA and OCA) on the physical, barrier, and antioxidant properties of collagen fiber–starch composite films. The results demonstrate that incorporating polyphenols significantly enhances the mechanical strength, water vapor barrier properties, and UV–visible light shielding capacity of the films, while simultaneously imparting remarkable antioxidant functionality. In particular, oxidized polyphenols (OTA and OCA) further improved film compactness, mechanical robustness, and thermal stability via quinone-mediated covalent cross-linking. From the perspective of collagen stabilization, the addition of polyphenols effectively reinforces the collagen fiber network through multiple molecular interactions—including hydrogen bonding, hydrophobic interactions, and covalent cross-linking—thereby enhancing the overall structural integrity and durability of the composite film. Compared with traditional chemical cross-linkers (e.g., glutaraldehyde) or physical modification methods, polyphenols achieve effective cross-linking while avoiding potentially toxic by-products and simultaneously provide additional antioxidant activity, highlighting their clear advantages for food-contact materials.

Furthermore, release kinetics studies revealed a distinct ethanol-concentration-dependent release behavior of the polyphenols, offering a theoretical and practical foundation for designing controlled-release active packaging systems tailored to specific food matrices, such as alcoholic beverages and fermented foods. Future research should focus on the following aspects: (1) quantitatively elucidating the contributions and kinetics of different cross-linking mechanisms in the polyphenol–collagen–starch ternary system; (2) evaluating the long-term effects of polyphenol-stabilized films on lipid oxidation, pigment retention, and microbial inhibition under realistic food-packaging conditions; (3) exploring the synergistic effects of polyphenols with other natural active components (e.g., essential oils and antimicrobial peptides) to develop integrated, multifunctional packaging systems; and (4) assessing the bioaccessibility and biosafety of the composite films through in vitro digestion and cellular assays to provide systematic support for their practical application.

## Figures and Tables

**Table 1:** Basic properties of composite films.

Treatment	WVP (g·mm/kPa·h·m^2^)	Water Solubility (%)	Opacity
Control	1.32 ± 0.03 ^b^	16.32 ± 0.19 ^a^	3.12 ± 0.05 ^a^
TA_0.05%_	1.28 ± 0.02 ^cd^	15.09 ± 0.20 ^b^	3.21 ± 0.10 ^a^
TA_0.075%_	1.26 ± 0.02 ^d^	15.13 ± 0.28 ^b^	3.41 ± 0.18 ^b^
TA_0.1%_	1.31 ± 0.03 ^bc^	14.92 ± 0.31 ^bc^	3.81 ± 0.14 ^c^
TA_0.125%_	1.41 ± 0.01 ^a^	14.53 ± 0.20 ^c^	3.88 ± 0.11 ^c^
Control	1.32 ± 0.03 ^b^	16.32 ± 0.19 ^a^	3.12 ± 0.05 ^a^
CA_0.05%_	1.26 ± 0.02 ^c^	14.81 ± 0.35 ^c^	3.16 ± 0.05 ^a^
CA_0.075%_	1.26 ± 0.01 ^c^	15.11 ± 0.18 ^c^	3.30 ± 0.11 ^a^
CA_0.1%_	1.34 ± 0.33 ^b^	15.63 ± 0.29 ^b^	3.34 ± 0.23 ^b^
CA_0.125%_	1.38 ± 0.01 ^a^	15.80 ± 0.23 ^b^	3.38 ± 0.11 ^b^
Control	1.32 ± 0.03 ^a^	16.32 ± 0.19 ^a^	3.12 ± 0.05 ^a^
OTA_0.05%_	1.05 ± 0.03 ^c^	14.37 ± 0.24 ^b^	4.49 ± 0.33 ^b^
OTA_0.075%_	1.08 ± 0.04 ^c^	14.33 ± 0.24 ^b^	4.61 ± 0.33 ^bc^
OTA_0.1%_	1.12 ± 0.05 ^c^	13.83 ± 0.34 ^b^	4.65 ± 0.28 ^bc^
OTA_0.125%_	1.26 ± 0.03 ^b^	14.16 ± 0.39 ^b^	4.96 ± 0.28 ^c^
Control	1.32 ± 0.03 ^a^	16.32 ± 0.19 ^a^	3.12 ± 0.05 ^a^
OCA_0.05%_	1.14 ± 0.01 ^d^	15.07 ± 0.52 ^b^	4.43 ± 0.15 ^b^
OCA_0.075%_	1.15 ± 0.03 ^cd^	14.54 ± 0.23 ^bc^	4.59 ± 0.25 ^b^
OCA_0.1%_	1.19 ± 0.02 ^bc^	14.42 ± 0.43 ^c^	4.64 ± 0.04 ^b^
OCA_0.125%_	1.20 ± 0.02 ^b^	14.14 ± 0.09 ^c^	4.90 ± 0.21 ^c^

Note: different superscript letters (a–d) denote significant differences (*p* < 0.05), while shared letters indicate no statistical difference (*p* > 0.05).

## Data Availability

The original contributions presented in this study are included in the article. Further inquiries can be directed to the corresponding authors.
